# Navigating Dichotomies and Dilemmas: Comment on "Dual Agency in Hospitals: What Strategies Do Managers and Physicians Apply to Reconcile Dilemmas Between Clinical and Economic Considerations?"

**DOI:** 10.34172/ijhpm.2022.6965

**Published:** 2022-05-21

**Authors:** Stephen Sirris

**Affiliations:** VID Specialized University, Oslo, Norway.

**Keywords:** Hybrid Professional Managers, Hospital, Dilemma, Reflection, Ethics

## Abstract

The complexity within healthcare typically surfaces in tensions between organizational efficiency and professional values when hospital managers and professionals face dilemmas between economic and clinical considerations. Waitzberg et al, based on interviews with managers and physicians working in Israeli and German hospitals, explore strategies to cope with such dilemmas to safeguard both financial sustainability and patient quality. This short commentary provides some follow-up questions in two ways. First, I highlight the importance of nuancing and hybridizing the dichotomies that lead to dilemmas in the hospital context. Second, I discuss how such dilemmas can be highlighted in systematic group reflection which in turn can inform decision-making.

## Complexity Triggers Dichotomies and Dilemmas

 Hospitals are increasingly challenged by greater demands for efficiency and quality through reforms and new technology. The article by Waitzberg et al^1^ pinpoints this central theme within health management. Their article explores a well-known phenomenon; the meeting of financial sustainability and clinical quality as experienced by hospital managers and physicians. Thus, they address a nexus of crucial issues for practitioners and researchers alike which adds to the relevance of their study. Priority-setting and well-founded decisions is a key challenge in hospitals. Beyond describing this complexity, their article shows how the gaps of dilemmas are bridged in everyday hospital work.

 Their interview study, with granulated, yet ambitious data, is distributed on five hospitals in two nations, Germany in Israel, with 33 interviews from Israel and 13 from Germany. Doing comparative research in organizational and management studies is demanding, however, this endeavour needs encouragement. On the national level, both countries share characteristics of healthcare system in finance as they are paid based on activity. A common denominator in both countries, in Germany from 2003 and Israel from 1990s, is that medical diagnoses are subjected to an activity-based financial system (diagnoses related group, DRG) to counter criticisms of high costs, low transparency, and long waiting lists. DRG estimated treatment costs on the basis of diagnosis, treatment, gender, age and so on. Since then, hospital budgets are financed with DRG coding and through block financing.

 The authors’ venture point is clearly stated; the dilemmas that are inherent in hospital practices and studied over a range of years in various literatures.^2^ A dilemma can shortly be described as a situation in which a difficult choice has to be made between at least two alternatives. Both are equally desirable and undesirable, they have positive as well as negative aspects and will have imperfect consequences.^3^ I will make some comments on the underlying issue in their article, first, that dichotomies lead to dilemmas, and second, how dilemmas can be dealt with. I particularly elaborate on balancing the demands of organizational efficiency, which is basically instrumental, with professional ethics, that in nature is normative.

## Nuancing and Hybridizing Dichotomies

 When studying tensions, inherent dichotomous pairs as coined by Waitzberg et al are managers-professionals, clinical-economic considerations, and comparisons between Germany-Israel. A wealth of literature is available on the linkage between professionalism and managerialism within the health sector.^4-7^ While earlier research pointed to the dichotomous nature of professionals versus managers and organizations, recent researchers have focused on how both professionals and managers handle diverse logics in their daily practice.^2,5,6^ In organizations, professionals encounter a corporative logic with management and governance because work specialization necessitates coordination and cooperation. However, departing from this dichotomy their study engages with and explores the overlaps and possible dilemmas at the intersections.

 Hybridity in organizations was initially described by Albert and Whetten.^8^ The concept was mainly relevant in situations where the public sector borrowed functional elements for the private sector. Today, the term hybrid is used in various ways, and a common problem is its use to describe an organization or management that does not fit established notions. Hybrid professional managers are individuals with a professional background who have moved into formal managerial roles and exercise leadership within their own professional group.^5,6^ Overall, hybridization implies a negotiating stance, compatibility, and blurred boundaries. Numerato et al^7^ note that professionalism and management are framed as contradictory in the work of actors. However, contextual and situated “analysis have concluded that interplay between professionalism and management results more often in coexistence, co-optation, mediation, negotiating, emerging and (strategic) adaptation rather than in clashes, hegemony and resistance.”

 Waitzberg et al explore the assumed dichotomies by pinpointing the distinctive contrasting pair of economic and clinical considerations. Their study underlines that there needs not be misalignment, rather there can be synergies that reduce cost and improve clinical quality. By making such claims, their article adds to the studies exploring how conflicting demands are situated and dealt with in various ways in the everyday work of managers and professionals. In the midst of mundane activities, decisions have to be made in order “to get the job done.”^2^ By providing data from particular cases, Waitzberg et al illustrates how this unfolds by tracing situations where these considerations are aligned and those situations where dilemmas exist and describe strategies to cope with dilemmas in decision-making.

## Coping With Dilemmas

 There is one overarching question: How do hospitals navigate dilemmas? This question is addressed with one contextual dimension in mind; activity-based payment related to treatment. The findings show that most often dilemmas are aligned, meaning quality in care and financial sustainability. Yet, there may also be conflicts – what are the strategies of actors in these cases? Waitzberg et al identify three main strategies; (1) reshaping management includes better planning and improvement of coding, and (2) reframing decision making involves working with averages and developing tool-kites for decision making, and (3) inter-professional teams. These are interesting findings that would benefit from being examined in other national contexts as well. Here the dichotomy between structure and agency surfaces. Even though the organizational players exercise some agency, as dual agents committed to patients’ safety and to the organization’s financial sustainability, the dilemmas emerge in choices and priorities in admission and treatment of patients.

 Hospitals are complex and hybrid organizations with various sets of tasks, missions and resources. As complex knowledge organizations,^9^ they entail different logics. The interest in practices related to the clinic, is a fruitful soil for dilemmas. This observation is the very point of departure for Waitzberg et al. Working with dilemmas thus invites exploring the alternatives at hand, stakeholders, interests, values and possible course of action and the agency involved.^3^ Dilemmas can at first sight be established as a dichotomy, however, they simultaneously allow for discovering and reflecting on subtle nuances. Since dilemmas occur in complexity and involves tensions, such nuances are not always obvious to all parties. Reflection is needed.

 Handling clinical and economic concerns in hospitals, points towards ethics. In a systematic review,^10^ organizational demands lead to moral distress for nurses that compromises their professional values of patient care. An overall value in healthcare from the professional point of view, is to put the patient first in terms of providing the best possible quality and safety. This mission indicates that hospitals do not exist to earn money, but to treat patients and hinder pain.^6,7^ Consequently, in the professional perspective, economy is a means to this end.^2^ If accepting these premises, and assisted by the notion of dichotomies, hospitals promote both normative values and rational goals.^9^ Waitzberg et al do focus the professional aspects of the hospital which certainly could be taken into account as the theme is dilemmas and decisions. Choice of action to realize these values and goals are made within particular frames consisting of several systems of norms and decisions. To which the article testifies, middle managers not the least are involved in many ethical and clinical dilemmas. Hitherto, it is crucial to provide sufficient information based on medical, economic, legal *and* ethical aspects.^3,10^ Hospitals depend on the competencies and time of the professional staff. A key managerial task is monitoring competencies and facilitate their development. Increased regulations can delimit the manoeuvring space by administrative systems, supervision and reporting. These demands can be on the expense of quality in treatment and care. This means that ethical considerations are very much present in this research context.

 Ethical reflection is not only be individual, but can also be organizationally grounded and facilitated.^3^ There seems to be dynamics between rational and ethical stances. Healthcare professionals are nurtured by the moral dimension of their professional ethics. Yet, values may conflict and must be mediated and negotiated. Hospitals are realized in the meeting between patients and treatment. How core values are contingent, is studied in a case study from a Norwegian hospital.^11^ This appears to a blind zone in the article which in tangible in its bias by lacking conflicts and not reconciling, which is also admitted by the authors (p. 10).

 The rational approach preferred by the authors is the search for tools applicable across contexts. An interesting point is that it is “clear treatment guidelines, instructions and information, that constitute a tool-kit for decisions-making. This provides criteria for using expensive materials and procedures, prioritizing.” This implies a standardization, “thus relieving them from having to weigh the different considerations based only on their own values and knowledge” (p. 8). However, it is very hard to avoid professional and managerial discretion. There are limits for instructions, as pointed out by the authors: it can undermine the flexibility needed, reduce identification of particularities and the autonomy.

 What, then is ethical reflection, how is it done, what are the results? I will point to one method, ethical reflection in systematic groups with facilitator.^12^ There are various stepwise models, like the CME-model (Centre for Medical Ethics, Oslo): (1) What is the ethical question? An ethical challenge may be defined as a situation where there is uncertainty or disagreement about what is right or good. (2) What are the facts? (3) Who are the stakeholders and what are their views? (4) Which organizational, professional and individual values are at stake? (5) Which guidelines and laws are at stake? (6) Which alternatives for action exist? (7) Conclusion. In their systematic literature review on the evaluation of clinical ethics support in mental healthcare, Hem et al found participants’ increased insight into moral issues, and improved cooperation between professions. However, it is uncertain if this led to better patient care. Nonetheless, in another study, the significance of participating in systematic ethics reflection groups was explored.^13^ Systematic reflection gave participants a common ground to understand challenges, promotes learning and mutual understanding covering daily challenges. A well-structured approach to discuss ethical challenges, makes challenging problematic concepts, attitudes and practices feasible. According to this study, it also facilitates “constructive disagreement and room for internal critique, less judgmental reactions and more reasoned approaches, and identification of potential for improvement and alternative courses of action.”

## Conclusion

 Waitzberg et al argue that dilemmas are a joint concern for several professions, and I might add, for managers as well. Their empirical study supports findings avoiding dichotomies as managers and physicians are not isolated group with opposite values and goals. This is evident when exploring specific dilemmas.^2,3,10-13^ However, reflection is skill that must be trained over time to aid decision-making in acute situations. If not, moral distress will very likely occur.^14^ Thus, an important follow-up of the article is competency building among healthcare professionals and supporting the staff in the deliberation of clinical ethics dilemmas and policy development.

## Ethical issues

 Not applicable.

## Competing interests

 Author declares that he has no competing interests.

## Author’s contribution

 SS is the single author of the paper.
